# Supplementary material for: Determination of Fumonisins in Grains and Poultry Feedstuffs in Croatia: A 16-Year Study
Source: Toxins (Basel). 2022 Jun 29;14(7):444. doi: 10.3390/toxins14070444 (PMC9318733; doi:10.3390/toxins14070444)
Supplement: Supplementary file 1 [file toxins-14-00444-s001.zip › toxins-1778015-supplementary.pdf]

toxins

Supplementary Materials: Determination of Fumonisins in Grains and Poultry Feedstuffs in Croatia: A 16-Year Study

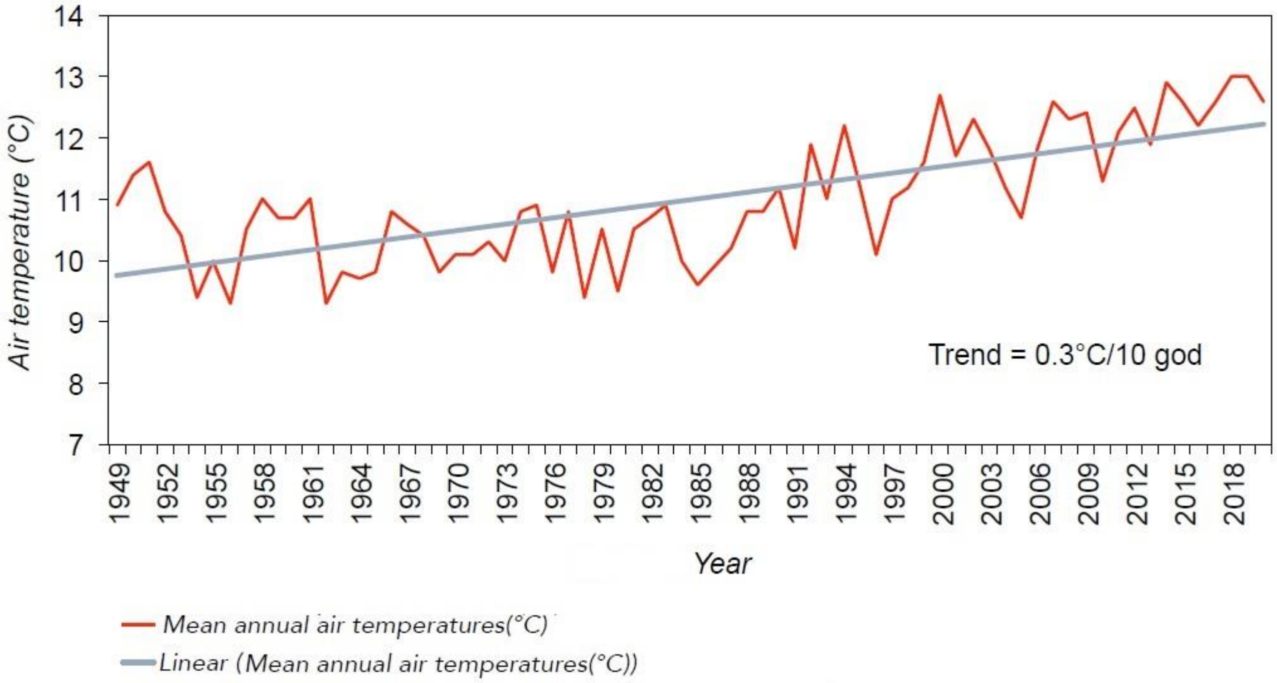

Figure S1. Trend of annual precipitation amounts in Croatia (measured in Zagreb-Maksimir in the period 1949 - 2020) [54].

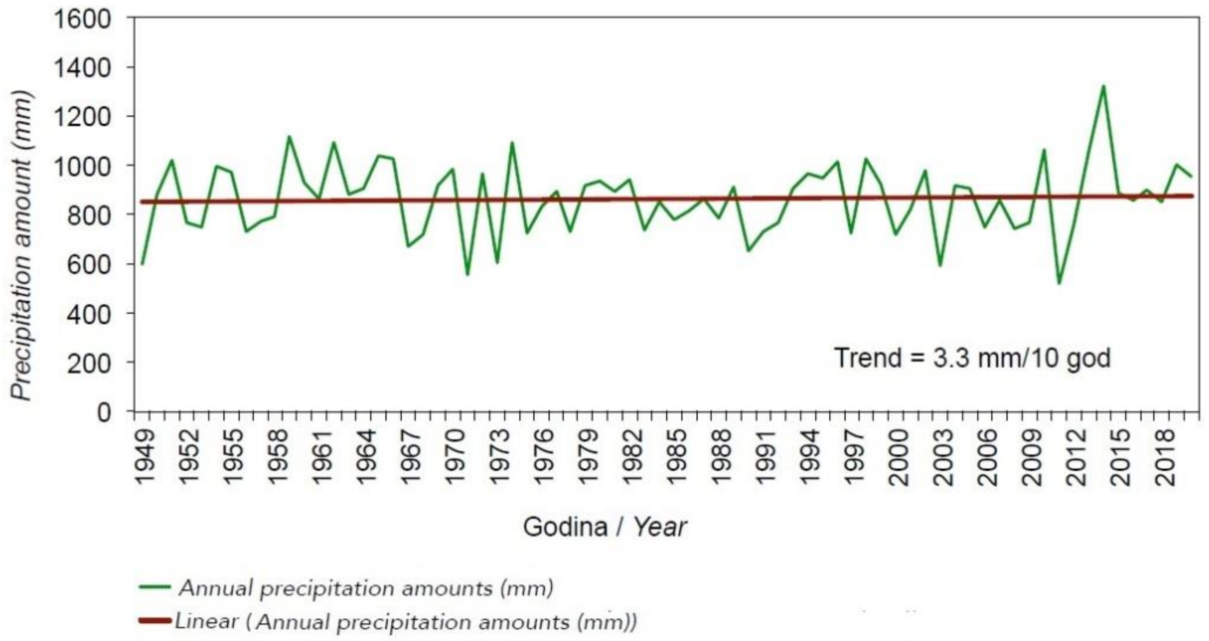

Figure S2. Trend of mean annual air temperature in Croatia (measured in Zagreb-Maksimir in the period 1949 - 2020) [54].

.

Table S1. Results on incidence of Fumonisins (FB1+FB2+FB3) in different Poultry Feed Samples from Croatia, analysed in the 16-year period (2006-2021).

**Year**		**Samples**			**Fumonisin (B1+B2+B3) (mg/kg)**			
	**Type**	**No**	**Pos**	**%pos**	**Min**	**Max**	**Mean**	**Med**
**2006**	Feed	23	21	91.3	0.01	2.14	0.42	0.10
	Broiler	12	11	91.7	0.01	2.14	0.46	0.10
	Laying Hens	3	3	100.0	0.01	0.35	0.12	0.01
	Uncategorized	8	4	50.0	0.10	1.53	0.56	0.37
**2007**	Feed	48	35	72.9	0.09	2.33	0.86	0.84
	Broiler	17	13	76.5	0.09	2.10	0.94	0.64
	Laying Hens	5	0	0.0	0.00	0.00	0.00	0.00
	Uncategorized	26	22	84.6	0.10	2.33	0.81	0.91
**2008**	Feed	54	37	68.5	0.10	2.79	0.72	0.44
	Broiler	5	3	60.0	0.72	1.67	1.12	0.98
	Laying Hens	5	3	60.0	0.10	2.79	1.37	1.23
	Uncategorized	44	31	70.5	0.10	1.85	0.61	0.39
**2009**	Feed	31	19	61.3	0.01	2.68	0.94	0.86
	Broiler	12	8	66.7	0.01	1.25	0.72	0.79
	Laying Hens	9	5	55.6	0.11	1.92	1.03	1.27
	Uncategorized	10	6	60.0	0.53	2.68	1.17	0.88
**2010**	Feed	29	17	58.6	0.06	0.89	0.34	0.15
	Broiler	12	10	83.3	0.06	0.89	0.35	0.14
	Laying Hens	6	0	0.0	0.00	0.00	0.00	0.00
	Uncategorized	11	7	63.6	0.10	0.76	0.34	0.15
**2011**	Feed	26	26	100.0	0.01	1.63	0.81	0.92
	Broiler	16	16	100.0	0.01	1.44	0.74	0.92
	Laying Hens	4	4	100.0	0.10	1.51	0.96	1.11
	Uncategorized	6	6	100.0	0.25	1.63	0.90	0.93
**2012**	Feed	12	10	83.3	0.26	1.37	0.81	0.88
	Broiler	6	5	83.3	0.26	1.37	0.86	0.88
	Laying Hens	5	4	80.0	0.26	0.99	0.67	0.72
	Uncategorized	1	1	100.0	1.07	1.07	1.07	1.07
**2013**	Feed	30	29	96.7	0.10	2.60	0.80	0.71
	Broiler	12	12	100.0	0.10	2.16	0.99	0.86
	Laying Hens	6	6	100.0	0.10	0.54	0.27	0.28
	Uncategorized	12	11	91.7	0.27	2.60	0.90	0.72
**2014**	Feed	23	21	91.3	0.11	2.29	0.81	0.60
	Broiler	11	10	90.9	0.20	2.29	1.00	0.70
	Laying Hens	6	5	83.3	0.11	1.75	0.62	0.30
	Uncategorized	6	6	100.0	0.39	1.27	0.66	0.56
**2015**	Feed	15	14	93.3	0.24	3.14	1.22	1.06
	Broiler	8	8	100.0	0.24	3.14	1.40	1.16
	Laying Hens	5	4	80.0	0.34	0.78	0.65	0.73
	Uncategorized	2	2	100.0	1.55	1.77	1.66	1.66
**2016**	Feed	8	8	100.0	0.38	5.72	2.63	3.06
	Broiler	4	4	100.0	1.26	3.34	2.72	3.15
	Laying Hens	3	3	100.0	0.38	5.72	2.35	0.94
	Uncategorized	1	1	100.0	3.10	3.10	3.10	3.10

**2017**	Feed	9	8	88.9	0.12	1.15	0.69	0.65
	Broiler	5	5	100.0	0.12	1.15	0.60	0.54
	Laying Hens	2	2	100.0	0.37	0.98	0.68	0.68
	Uncategorized	2	1	50.0	1.15	1.15	1.15	1.15
**2018**	Feed	10	9	90.0	0.02	1.06	0.37	0.27
	Broiler	5	5	100.0	0.02	1.06	0.41	0.10
	Laying Hens	3	3	100.0	0.27	0.46	0.36	0.34
	Uncategorized	2	1	50.0	0.19	0.19	0.19	0.19
**2019**	Feed	14	14	100.0	0.10	1.02	0.51	0.40
	Broiler	6	6	100.0	0.10	1.02	0.63	0.65
	Laying Hens	4	4	100.0	0.10	0.10	0.10	0.10
	Uncategorized	4	4	100.0	0.15	1.02	0.76	0.93
**2020**	Feed	8	6	75.0	0.10	2.58	1.31	1.23
	Broiler	6	4	66.7	0.10	2.58	1.35	1.37
	Laying Hens	2	2	100.0	1.23	1.23	1.23	1.23
	Uncategorized	0	0	0.0	0.00	0.00	0.00	0.00
**2021**	Feed	5	5	100.0	0.40	9.64	2.34	0.53
	Broiler	3	3	100.0	0.49	0.66	0.56	0.53
	Laying Hens	2	2	100.0	0.40	9.64	5.02	5.02
	Uncategorized	0	0	0.0	0.00	0.00	0.00	0.00

No - number; Pos - positive; %pos - percentage of positive; Min - minimum; Max - maximum; Mean - average value; Med - median value.

Table S2. Additional results of analysed samples. Results on incidence of Fumonisins (FB1+FB2+FB3) in different Poultry Feed Samples from Croatia, analysed in the 16-year period (2006-2021).

Year	Samples				Fumonisin (B1+B2+B3) (mg/kg)			
	Type	No	Pos	%pos	Min	Max	Mean	Med
**2006**	Grains	17	12	70.6	0.01	2.52	1.07	0.77
	Maize	12	9	75.0	0.01	2.52	0.97	0.42
	Wheat	3	1	33.3	0.20	0.20	0.20	0.20
	Soybean	2	2	100	0.10	0.77	0.44	0.44
**2007**	Grains	43	28	65.1	0.10	2.81	1.02	0.98
	Maize	30	21	70.0	0.10	2.81	1.11	1.00
	Wheat	9	6	66.7	0.10	1.72	0.82	0.94
	Soybean	4	1	25.0	0.28	0.28	0.28	0.28
**2008**	Grains	65	33	50.8	0.10	2.57	0.48	0.24
	Maize	46	24	52.2	0.10	2.57	0.55	0.25
	Wheat	9	3	33.3	0.12	0.31	0.20	0.16
	Soybean	10	6	60.0	0.10	0.81	0.31	0.26
**2009**	Grains	39	24	61.5	0.10	1.67	0.70	0.66
	Maize	20	13	65.0	0.10	1.47	0.70	0.62
	Wheat	10	4	40.0	0.10	0.87	0.47	0.46
	Soybean	9	7	77.8	0.20	1.67	0.83	0.78
**2010**	Grains	67	57	85.1	0.01	5.85	1.00	0.30
	Maize	55	51	92.7	0.01	5.85	1.00	0.25
	Wheat	4	2	50.0	0.48	0.67	0.58	0.58
	Soybean	8	4	50.0	0.01	2.76	1.18	0.78
**2011**	Grains	28	19	67.9	0.01	1.95	0.66	0.43
	Maize	14	12	85.7	0.01	1.95	0.65	0.39
	Wheat	7	4	57.1	0.05	1.80	1.14	1.36
	Soybean	7	3	42.9	0.05	0.60	0.23	0.05
**2012**	Grains	55	55	100	0.01	7.31	0.75	0.56
	Maize	44	44	100	0.01	7.31	0.80	0.54
	Wheat	6	6	100	0.02	1.30	0.37	0.07
	Soybean	5	5	100	0.27	1.03	0.83	0.95
**2013**	Grains	30	30	100	0.07	2.95	0.93	0.73
	Maize	25	25	100	0.07	2.95	0.84	0.64
	Wheat	3	3	100	0.96	2.87	1.59	0.96
	Soybean	2	2	100	0.29	1.72	1.01	1.01
**2014**	Grains	27	24	88.9	0.03	9.30	2.46	1.42
	Maize	15	12	80.0	0.03	9.30	3.33	2.76
	Wheat	8	8	100	0.31	4.12	1.79	1.22
	Soybean	4	4	100.0	0.31	1.39	0.85	0.86
**2015**	Grains	36	35	97.2	0.10	6.42	1.68	1.33
	Maize	27	26	96.3	0.12	3.47	1.58	1.34
	Wheat	4	4	100.0	0.10	3.53	1.10	0.39
	Soybean	5	5	83.3	0.21	6.42	2.63	1.68
**2016**	Grains	18	18	100.0	0.10	3.47	0.75	0.14
	Maize	11	11	100.0	0.10	2.99	0.41	0.10
	Wheat	3	3	100.0	0.11	3.47	1.54	1.05
	Soybean	4	4	100.0	0.13	3.15	1.10	0.57
**2017**	Grains	17	15	88.2	0.28	2.84	1.08	0.82

	Maize	10	9	90.0	0.37	2.84	1.14	0.72
	Wheat	4	3	75.0	0.28	0.91	0.61	0.65
	Soybean	3	3	100.0	0.82	2.19	1.37	1.11
**2018**	Grains	50	46	92.0	0.01	4.15	0.48	0.20
	Maize	35	35	100.0	0.04	4.15	0.46	0.20
	Wheat	9	5	55.6	0.04	0.90	0.27	0.13
	Soybean	6	6	100.0	0.01	1.21	0.42	0.05
**2019**	Grains	32	9	28.1	0.10	3.44	0.97	0.20
	Maize	17	5	29.4	0.10	3.44	1.48	0.20
	Wheat	8	2	25.0	0.20	0.50	0.35	0.35
	Soybean	7	2	28.6	0.20	0.50	0.35	0.35
**2020**	Grains	34	30	88.2	0.09	3.22	1.46	1.48
	Maize	25	24	96.0	0.09	3.22	1.55	1.71
	Wheat	4	2	50.0	0.20	3.22	1.14	0.20
	Soybean	4	3	75.0	0.20	0.79	0.40	0.20
**2021**	Grains	30	27	90.0	0.04	22.23	1.50	0.50
	Maize	20	19	95.0	0.04	22.23	1.84	0.54
	Wheat	5	3	60.0	0.05	1.05	0.52	0.47
	Soybean	5	5	100.0	0.13	0.71	0.34	0.17

No - number; Pos - positive; %pos - percentage of positive; Min - minimum; Max - maximum; Mean - average value; Med - median value.

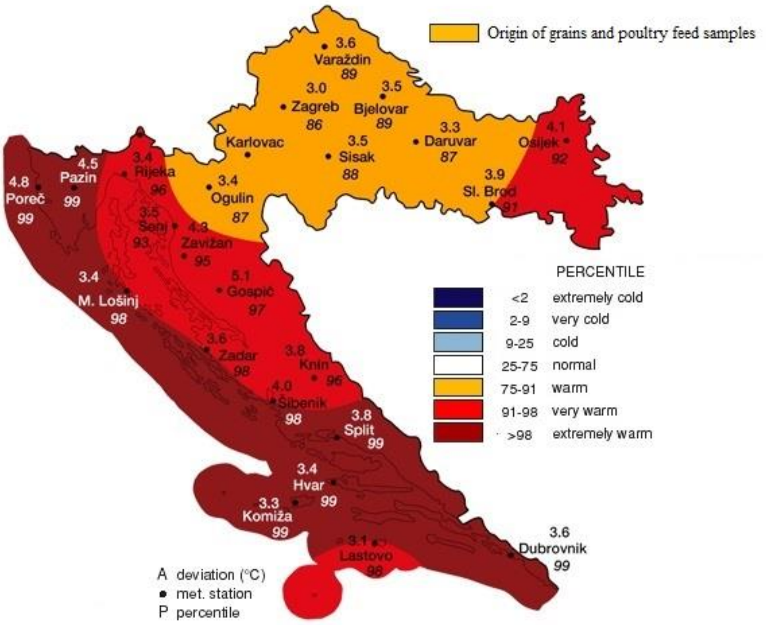

Figure S3. Geographical origin of analysed samples (adapted from the official Croatian Metrological website with data on evaluation of climate conditions in Croatia (www.meteo.hr). Croatia is situated in southeast Europe adjoining the Mediterranean, central and southeast Europe. It lies between latitude 42° 23′ and 46° 33′ north, and between longitude 13° 30′ and 19° 27′ east. Geographical coordinates of this sampling area are following north (46° 33' N, 16° 22' E), west (45° 28' N, 14° 54' E), south (44° 50' N, 15° 21' E), and east (45° 44' N, 18° 26' E).
